# Hydroxy-Selenomethionine Supplementation During Gestation and Lactation Improve Reproduction of Sows by Enhancing the Antioxidant Capacity and Immunity Under Heat Stress Conditions

**DOI:** 10.3390/antiox14050525

**Published:** 2025-04-27

**Authors:** Juan Wang, Hua Sun, Zhe Peng, Shao-Qing Wang, Yi-Qin Yan, Wei-Cai Luo, Ren-Gui Yang, Wei-Cheng Bei, Lv-Hui Sun, Jia-Cheng Yang

**Affiliations:** 1 State Key Laboratory of Agricultural Microbiology, Hubei Hongshan Laboratory, Key Laboratory of Smart Farming Technology for Agricultural Animals of Ministry of Agriculture and Rural Affairs, Frontiers Science Center for Animal Breeding and Sustainable Production, College of Animal Science & Technology and College of Veterinary Medicine, Huazhong Agricultural University, Wuhan 430070, China; wangjuan2021@webmail.hzau.edu.cn (J.W.); pxzhe@webmail.hzau.edu.cn (Z.P.); wsq1999@webmail.hzau.edu.cn (S.-Q.W.); yiqinyan@webmail.hzau.edu.cn (Y.-Q.Y.); luoweicai@webmail.hzau.edu.cn (W.-C.L.); yrg5068@trsgroup.cn (R.-G.Y.); beiwc@mail.hzau.edu.cn (W.-C.B.); 2 Inner Mongolia Academy of Agriculture and Animal Husbandry Science, Hohhot 010031, China; sunhua564116908@163.com

**Keywords:** sow, piglet, performance, hydroxy-selenomethionine, selenoprotein, heat stress

## Abstract

The objective of this study was to determine whether hydroxy-selenomethionine (OH-SeMet) exerts better protective effects on sows against heat stress than sodium selenite (SeNa) or seleno-yeast (SeY). A total of 60 sows (Landrace × Yorkshire) were randomly allocated into the three groups and fed a base diet supplemented with SeNa, SeY, or OH-SeMet at 0.3 mg Se/kg under a heat stress condition for a reproductive cycle. Compared to SeNa or SeY, OH-SeMet could more effectively sustain offspring growth performance, as evidenced by an increased number of live-born piglets, higher litter weight at day 21, and greater litter body weight gain from days 1 to 21. OH-SeMet was more effective in supporting endogenous redox systems, as shown by enhanced levels of TXNRD and GSH and reduced levels of GSSG in the serum of sows, improved T-AOC, TXNRD, and GSH alongside decreased MDA and GSSG in the serum of piglets, and heightened T-AOC in the jejunum of piglets. Furthermore, among the two tested organic Se sources, OH-SeMet was more effective than SeY in regulating immune responses compared to SeNa. OH-SeMet reduced inflammation-related markers CRP, HP, MAP, LPS, IL-1β, IL-6, and TNF-α, some or all of which were reduced in the serum of sows and their offspring. In addition, OH-SeMet also showed reduced glucose, TG, and NEFA levels, along with elevated insulin levels in the serum of sows. Correspondingly, among the two organic forms of Se, particularly those sows fed OH-SeMet showed better gut protection for the sows’ offspring, as indicated by a reduced crypt depth and increased villus height/crypt depth ratio in the duodenum, jejunum, and ileum than those fed SeNa. Specifically, compared to SeNa or SeY, OH-SeMet upregulated the expression of selenoproteins (*GPX6*, *TXNRD3*, GPX4, and SELENON), the tight junction protein (ZO-1), and host defense peptide gene (*pBD1*, *pBD2*, *pBD3*, *NPG3*, *NPG4*), along with downregulating levels of inflammation factor (IL-1β, IL-6 and TNF-α) and pro-apoptotic factor (P53) in the jejunum of piglets. Taken together, OH-SeMet more effectively mitigated the adverse effects induced by heat stress in sows and their offspring.

## 1. Introduction

Heat stress is one of the most major environmental stressors that is detrimental to animal husbandry worldwide [1,2]. Swine are particularly sensitive to hot environments due to a lack of functional sweat glands [3]. This is even worse for sows during gestation and lactation, with high metabolic load due to fetal growth, milk production, and litter-rearing, which increases individual sensitivity [4]. Heat stress causes a series of adaptive behavioral and metabolic changes [5], including reduced feed intake, increased embryo mortality and stillborn piglets, lower farrowing rates, smaller litter sizes, and loss of body condition during pregnancy and lactation in sows [6]. These metabolic disturbances have been attributed to the disturbance of redox balance, causing lipid peroxidation and oxidative damages to proteins, DNA, and other biological molecules, inhibition of immune function, and metabolic disorders [7,8].

Selenium (Se) is an essential nutrient for humans and animals, with crucial functions in the antioxidant system, immunity, and detoxification [9,10]. These metabolic functions of Se have been attributed mainly to its presence in 25 selenoproteins as the 21st amino acid, selenocysteine (SeCys, U), in pigs. Previous studies have shown that plasma Se concentration decreases during gestation [11]; and dietary supplementation of Se to gestating and lactating sows can effectively protect against the adverse effects induced by metabolic and environmental stress on reproductive performance, milk quality, and the body weight of weaned pigs [12,13,14]. These protective actions of Se against stressors are attributed to adequate dietary Se supplementation and an optimal Se status in the body, resulting in the improvement of antioxidant capacity, and modulation of the inflammatory response and immune functions [15].

For the purpose of avoiding an Se deficiency and fulfilling the nutritional requirements of livestock, it is standard practice to supplement the diet with Se as either inorganic (sodium selenite, SeNa) or organic Se, the latter of which is widely used due to its higher bioavailability due to the presence of selenomethionine (SeMet) [15,16]. Organic forms of Se can be found as seleno-enriched yeast (SeY) or in pure forms of SeMet (i.e., hydroxy-selenomethionine, OH-SeMet). The primary advantage of dietary Se supplementation in the form of SeMet over inorganic Se sources is that a portion of SeMet follows the methionine pathway and is stored in the protein pool, while the rest is metabolized like other selenium sources to meet immediate selenoprotein needs [16,17,18,19]. Increased tissue reserves of Se can improve the resistance of animals to environmental stress and metabolic diseases and represent a pivotal strategy to help the animals deal with relevant stressors affecting production [16,20]. SeY contains, according to its technical specifications, 97% of organic Se in which ≥63% of the total Se is SeMet. However, it has been reported that the SeMet content of these products does not always comply with the minimum 63% and more recently it has been reported that the inorganic content is often higher than 3% [21,22,23]. This results in a non-reliable product, not able to optimize the Se status of animals. Pure forms of organic Se, such as OH-SeMet, on the other hand, provide consistently ≥ 98% of the total Se as OH-SeMet [24,25,26,27].

Thus far, studies have shown that OH-SeMet displayed functional benefits compared to SeNa and SeY in dairy cows [28,29], chickens [17,30,31,32], and growing pigs [33,34]. Moreover, it has been proven that OH-SeMet increases the Se deposition in tissues and upregulates several selenoproteins’ gene expression in animals, compared to SeNa and SeY [17]. Since OH-SeMet enhances selenoprotein levels more effectively than SeNa and SeY, it plays a crucial role in redox balance and immune regulation [35,36]. However, research on its effects under heat stress conditions, particularly in gestating and lactating sows, remains limited. This study hypothesizes that OH-SeMet may provide additional protection and benefits to sows experiencing physiological metabolic and heat stress. Therefore, this study was conducted to (1) determine whether OH-SeMet can better protect sows and piglets during gestation and lactation under heat stress conditions compared to SeNa or SeY; (2) explore the relevant molecular biology of such a process.

## 2. Materials and Methods

### 2.1. Animals, Treatments, and Sample Collection

The animal protocol of the present study was approved by the Institutional Animal Care and Use Committee of Huazhong Agricultural University, China. The ethical approval code was HZAUSW-2024-0030. In total, 60 sows (Landrace × Yorkshire) were assigned to 1 of the 3 treatment groups (*n* = 20 pens/group, 1 sow/pen) on gestation day 1. Animals were allocated based on their parity, backfat thickness, and body weight. Sows were fed a basal diet (Table S1) with 0.3 mg Se/kg added as SeNa, SeY, or OH-SeMet throughout the whole gestation and lactation. Following standard farm protocols, sows were feed-restricted during gestation to control body weight and prevent excessive fat accumulation that may have impaired reproductive performance, while their feed intake was gradually increased during lactation to protect gastrointestinal health and reduce the risk of metabolic disorders caused by sudden dietary changes [37,38]. Specifically, the sows were feed-restricted to 2.4, 2.0, 3.0, and 3.2 kg/day during the days 1–28, 29–84, 85–98, and 99–114 of gestation, respectively, and 2.0, 2.5, 3.0, 4.0, 5.0, 6.0, and 7.0 kg/day during days 1, 2, 3, 4, 5, 6, and 7–21 of lactation, respectively. The animal feeding trial was carried out between July and November of 2022 in Zhangzhou city, Fujian province of China, which is a period with often high temperatures and humidity—values which are presented in Figure S1. The average daily temperature in the sows’ house was 27.1 °C (ranging from 22.9 to 33.0 °C; Figure S1A), and the average relative humidity was 82.7% (ranging from 50.8 to 94.9%; Figure S1B) throughout the experiment. The sows were immunized with the porcine epidemic diarrhea (PED) and transmissible gastroenteritis coronavirus (TGEV) vaccines on days 85 of gestation [37]. The body weight, backfat thickness, weaning-to-estrus interval of sows, litter size, total born alive, stillborn, mummies, birth weight, weight gain, diarrhea, and mortality of progeny were recorded [39]. Colostrum and milk at day 14 of lactation and blood at day 107 of gestation and day 14 of lactation of sows, and blood at day 14 and 21 of piglets were harvested from the jugular vein for relative analysis. The piglets were weaned at lactation day 21, and 10 piglets from each treatment were sacrificed by electronarcosis, small intestinal and muscle samples were taken, and they then were stored at −80 °C for later analysis.

### 2.2. Milk Composition and Redox Parameter Analysis

The milk content, including fat, protein, lactose, and nonfat solids, was determined by an automated analyzer (Fossomatic^TM^ 7 somatic cell detector, FOSS, Hilleroed, Denmark) [40]. The concentrations of malondialdehyde (MDA), protein carbonyl (PC), glutathione (GSH), and oxidized glutathione (GSSG), and the activities of the total antioxidant capacity (T-AOC) and glutathione peroxidase (GPX) were measured by the specific assay kits of A003-1-2, A087-1-2, A006-2-1, A061-1-2, A015-2-1, and A005-1-2, respectively (Nanjing Jiancheng Bioengineering Institute, Nanjing, China). The activity of thioredoxin reductase (TXNRD) was determined by a specific assay kit (BW11) from the Suzhou Comin Biotechnology company of China [26]. The protein concentrations were measured by a bicinchoninic acid assay [41].

### 2.3. Serum Biochemical, Immunoglobulin, and Histological Analyses

The serum concentrations of glucose, total cholesterol (TC), triglyceride (TG), and non-esterified fatty acids (NEFAs) were measured using colorimetric assay kits, while insulin and lipopolysaccharides (LPSs) were determined using ELISA kits, all according to the manufacturer’s instructions (Nanjing Jiancheng Bioengineering Institute, China). The kit catalog numbers were as follows: A154-1-1 (glucose), A111-1-1 (TC), A110-1-1 (TG), A042-2-1 (NEFA), H203-1-1 (insulin), and H255-1-2 (LPS). The serum concentrations of C-reactive protein (CRP), haptoglobin (HP), pig major acute phase protein (MAP), cortisol, interleukins (IL)-1β, IL-6, IL-10, tumor necrosis factor-α (TNF-α), and immunoglobulins (IgA, IgG, IgM) were measured using ELISA kits (Cusabio Biotech Co., Ltd., Wuhan, China), following the manufacturer’s instructions. The kit catalog numbers were as follows: CRP (CSB-E08163p), HP (CSB-E13424p), MAP (CSB-E13425p), cortisol (CSB-E06811p), IL-1β (CSB-E06782p), IL-6 (CSB-E06786p), IL-10 (CSB-E06779p), TNF-α (CSB-E16980p-IS), IgA (CSB-E13234p), IgG (CSB-E06804p), and IgM (CSB-E06805p). The PED and TGEV antibody titers were measured by the specific ELISA Kits from Harbin National Engineering Research Center of Veterinary Biologics Corp (Harbin, China).

### 2.4. Histological Analyses

Histopathological analysis of the small intestine tissue (including duodenum, jejunum, and ileum) was carried out as previously described [15]. Briefly, intestine tissue was fixed in 10% neutral buffered formalin, embedded in paraffin, sectioned at 5 μm, and then stained with hematoxylin and eosin for morphological analysis. Histomorphometry measurements of intestine including villus height (µm), crypt depth (µm), and villus-to-crypt ratios (calculated as villus height/crypt depth) were obtained using Image J software (Version 1.51).

### 2.5. Real-Time q-PCR and Western Blot Analyses

Real-time q-PCR analyses of the jejunum samples were performed as previously described [9,42]. The primers were designed by Primer Express 3.0 (Applied Biosystems, Thermo Fisher scientistic, Waltham, MA, USA) and presented in Table S2. The relative mRNA expression level of target genes relative to the housekeeping gene (β-actin) was analyzed by the 2^−ddCT^ method. Western blot analyses of the jejunum samples were conducted as previously described [43]. The primary antibodies used for each gene are presented in Table S3. All protein levels were normalized to that of the housekeeping protein β-actin, and densitometry quantification of the Western blotting bands was performed using ImageJ software [44]. The relative density of the protein band was quantified using the Alpha-Imager 2200 system (Alpha Innotech, San Leandro, CA, USA).

### 2.6. Statistical Analysisher

Statistical analysis was performed using JMP Pro (version 17). The data are presented as the mean ± SE. Before data analysis, univariate outlier removal was conducted using Robust Fit Outliers, applying the K-Sigma fix at 3.0 Huber. After addressing outliers, ANOVA was run, followed by an analysis of least squares means (LSMeans) differences using Tukey’s test. Labeled means within the same plot without a common lowercase letter indicate significance at *p* < 0.05; a common uppercase letter indicates a significance level of 0.05 ≤ *p* < 0.10. It is important to note that since the qPCR data did not follow a normal distribution, the data were log-transformed before analyzing the least squares means (LSMeans) differences to assume a normal distribution.

## 3. Results

### 3.1. Growth Performance

All sows consumed the full daily ration due to the feed restriction during both gestation and lactation, ensuring consistent dietary intake and standardized management across all treatment groups. As shown in Table 1, sows supplemented with different Se sources exhibited similar body weight and backfat thickness during gestation and lactation stages, as well as weaning-to-estrus interval days. For piglets, compared to SeY, dietary supplementation with OH-SeMet increased (*p* < 0.05) the number of piglets remaining alive at 21d, and decreased diarrhea rate during the lactation period (0–21d), with SeNa showing intermediate values. In addition, compared to SeNa or SeY, dietary supplementation with OH-SeMet increased (*p *< 0.05) the litter body weight at day 21 and increased (*p *< 0.05) litter body weight gain during days 1–21 of lactation.

### 3.2. Redox Status in Sows and Piglets

**Figure 1:**
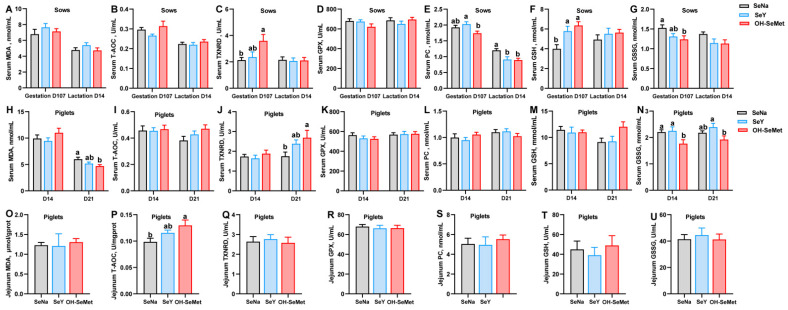
Effect of the three forms of Se on the redox status of sows and piglets. MDA, T-AOC, TXNRD, GPX, PC, GSH, and GSSG concentration or activity in serum of sows (**A**–**G**) and piglets (**H**–**N**), and in the jejunum of piglets (**O**–**U**). The values are the means ± SE, *n* = 19–20 in Figures (**A**–**N**), and *n* = 10 in Figures (**O**–**U**). Labeled means within the same plot without a common letter differ, *p* < 0.05. SeNa, basal diet supplemented with 0.3 mg Se/kg as sodium selenite; SeY, basal diet supplemented with 0.3 mg Se/kg as seleno-yeast; OH-SeMet, basal diet supplemented with 0.3 mg Se/kg as hydroxy-selenomethionine.

The effects of the different forms of Se on the redox status in sows are shown in Figure 1A–G. Compared to SeNa, both SeY and OH-SeMet enhanced (*p* < 0.05) the GSH concentration in serum at gestation day 107 of sows and resulted in a significantly (*p* < 0.05) lower concentration of PC in the serum at lactation day 14 of sows. OH-SeMet enhanced (*p* < 0.05) TXNRD activity and reduced the concentration of GSSG in serum at gestation day 107 of sows, with SeY showing intermediate values. Furthermore, compared to SeY, OH-SeMet resulted in a significantly (*p* < 0.05) lower concentration of PC in the serum at gestation day 107 of sows. In terms of the redox status of serum in piglets (Figure 1H–N), as compared with SeNa, OH-SeMet resulted in a significantly (*p* < 0.05) lower concentration of MDA in the serum of 21-day-old piglets and GSSG levels in 14-day-old piglets, while it also induced higher TXNRD activity in the serum of 21-day-old piglets. Meanwhile, SeY showed intermediate values of MDA and T-AOC between OH-SeMet and SeY. Furthermore, OH-SeMet resulted in a significantly (*p* < 0.05) lower concentration of GSSG in the serum of 21-day-old piglets compared with SeY, with SeNa showing equal and intermediate values in the serum of 21-days-old piglets. Lastly, regarding the redox status of jejunum in piglets, as shown in Figure 1O–U, OH-SeMet significantly increased (*p *< 0.05) T-AOC activity in the jejunum of 21-day-old piglets compared to SeNa, with SeY showing intermediate values.

### 3.3. Glucose- and Lipids-Related Index in Serum of Sows and Milk Composition

**Figure 2:**
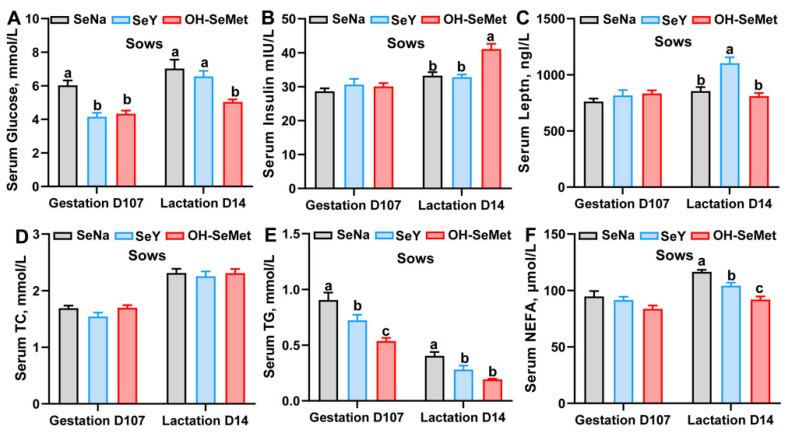
Effect of the three forms of Se on the glucose (**A**), insulin (**B**), leptin (**C**), TC (**D**), TG (**E**), and NEFA (**F**) concentration in the serum of sows. The values are the means ± SE, *n* = 19–20. Labeled means within the same plot without a common letter differ, *p* < 0.05. TC, total cholesterol; TG, triglycerides; NEFA, non-esterified fatty acids; SeNa, basal diet supplemented with 0.3 mg Se/kg as sodium selenite; SeY, basal diet supplemented with 0.3 mg Se/kg as seleno-yeast; OH-SeMet, basal diet supplemented with 0.3 mg Se/kg as hydroxy-selenomethionine.

The effects of the different forms of Se on the glycolipid metabolism-related indexes in serums of sows are shown in Figure 2A–F. Both OH-SeMet and SeY significantly reduced (*p* < 0.05) serum glucose levels at gestation day 107, NEFA levels at lactation day 14, and TG concentrations at both time points compared to SeNa, with OH-SeMet showing the lowest values. Furthermore, Only OH-SeMet decreased (*p* < 0.05) the concentration of glucose at lactation day 14 compared to SeNa. Additionally, OH-SeMet significantly increased (*p* < 0.05) insulin concentrations and SeY significantly elevated (*p* < 0.05) leptin concentrations at lactation day 14 compared to SeNa. In terms of milk composition, the different Se sources did not influence the milk composition of fat, protein, lactose and nonfat solids (Figure S2).

### 3.4. Immune Status of Sows and Piglets

**Figure 3:**
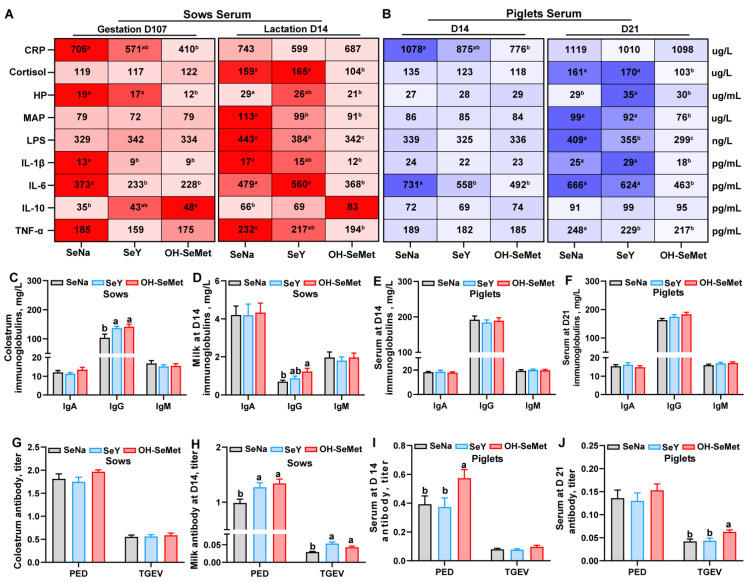
Effects of the three forms of selenium (Se) on inflammation-related indices (**A**,**B**), immunoglobulins (**C**–**F**), and antibody levels (**G**–**J**) in sows and piglets were evaluated. Inflammation-related indices, including CRP, cortisol, HP, MAP, LPS, IL-1β, IL-6, IL-10, and TNF-α were measured in the serum of sows and piglets. Immunoglobulins, including IgA, IgG, and IgM, were measured in the milk of sows and the serum of piglets. Antibodies, including PED and TGEV titers, were assessed in the milk of sows and the serum of piglets. The maximum and minimum values in Figure (**A**,**B**) share the darkest and lightest colors, respectively, while intermediate values transition gradually based on their magnitude. The values are the means or means ± SE, *n* = 19–20. Labeled means within the same plot without a common letter differ, *p* < 0.05. IgA, Immunoglobulin A; IgG, Immunoglobulin G, IgM, Immunoglobulin M; PED, porcine epidemic diarrhea; TGEV, transmissible gastroenteritis virus; CRP, C-reactive protein; HP, haptoglobin; MAP, pig major acute phase protein; LPS, lipopolysaccharide; SeNa, basal diet supplemented with 0.3 mg Se/kg as sodium selenite; SeY, basal diet supplemented with 0.3 mg Se/kg as seleno-yeast; OH-SeMet, basal diet supplemented with 0.3 mg Se/kg as hydroxy-selenomethionine.

The effects of the different forms of Se on the inflammation-related indices in the serum of sows and piglets are shown in Figure 3A,B. In sows, as compared with SeNa, both OH-SeMet and SeY reduced (*p* < 0.05) the concentrations of IL-1β and IL-6 at gestation day 107, as well as MAP and LPS at lactation day 14. Only OH-SeMet reduced the concentrations of CRP and HP while increasing IL-10 levels at gestation day 107. In addition, only OH-SeMet decreased the concentrations of, HP, IL-1β, IL-6, and TNF-α at lactation day 14. In contrast, SeY exhibited intermediate concentrations of CRP and IL-10 on gestation day 107 and of HP, IL-1β, and TNF-α on lactation day 14. For piglets, compared with SeNa, both SeY and OH-SeMet reduced the IL-6 concentrations in serum at day 14 and LPS concentrations in serum at day 21, while only OH-SeMet reduced the CRP concentrations in serum at day 14 and cortisol, MAP, and IL-6 concentrations in serum at day 21. Furthermore, SeY increased the HP concentrations in serum at day 21 compared with SeNa, with no changes in OH-SeMet. In terms of immunoglobulins concentrations in sow milk and piglet serum (Figure 3C–F), compared with SeNa, both SeY and OH-SeMet increased (*p* < 0.05) the IgG concentrations in the colostrum of sows, while only OH-SeMet enhanced IgG concentrations in the milk of sows at day 14. However, no significant difference was observed in immunoglobulin levels in piglet serum. Regarding the antibody level in the serum of sows (Figure 3G–J), both SeY and OH-SeMet enhanced (*p* < 0.05) the PED and TGEV titer in the milk of sows at day 14, while only OH-SeMet enhanced (*p* < 0.05) PED titer in the serum of piglets at day 14 and TGEV titer in the serum of piglets at day 21.

### 3.5. Small Intestine Morphology of the Piglets

**Figure 4:**
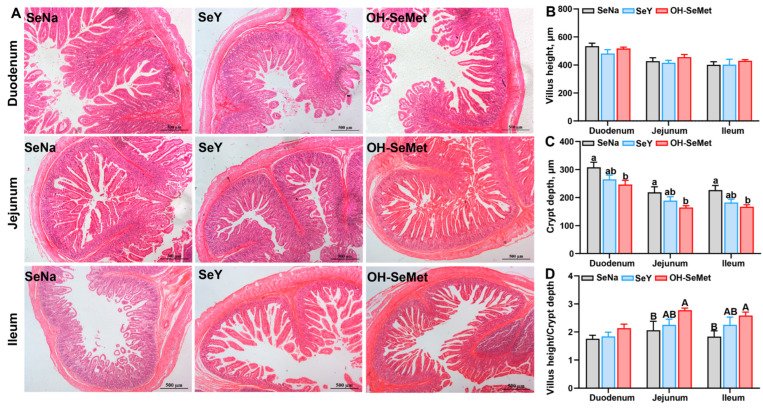
Effects of the three forms of Se on histology of the intestine (**A**), villus height (**B**), crypt depth (**C**), and villus height/crypt depth ratio (**D**) in piglets. The values are the means ± SE, *n* = 5. Labeled means within the same plot without a common lowercase letter indicate significance at *p* < 0.05; a common uppercase letter indicates a significance level of 0.05 ≤ *p* < 0.10. SeNa, basal diet supplemented with 0.3 mg Se/kg as sodium selenite; SeY, basal diet supplemented with 0.3 mg Se/kg as seleno-yeast; OH-SeMet, basal diet supplemented with 0.3 mg Se/kg as hydroxy-selenomethionine.

The small intestinal histology results are presented in Figure 4. The morphology results showed that different Se sources seemed to have no influence on the villus height. Only OH-SeMet reduced (*p *< 0.05) the crypt depth in the duodenum, jejunum, and ileum, with SeY showing intermediate values. Additionally, only OH-SeMet showed a trend toward increased (*p* < 0.10) intestinal villus to crypt ratios in jejunum and ileum, with SeY displaying intermediate values.

### 3.6. Expression of Selenoprotein, Tight Junction, Host Defense Peptide-Related, Cytokines, and Apoptosis Genes at mRNA Levels in the Jejunum of Piglets

**Figure 5:**
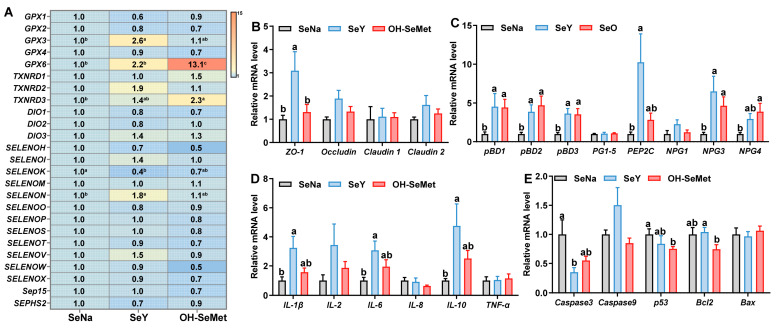
Effects of the three forms of Se on mRNA levels of selenoprotein (**A**), tight junction (**B**), host defense peptide-related (**C**), cytokines (**D**) and apoptosis genes (**E**) in the jejunum of piglets. The values are the means ± SE, *n* = 8–10. Labeled means within the same plot without a common letter differ, *p* < 0.05. SeNa, basal diet supplemented with 0.3 mg Se/kg as sodium selenite; SeY, basal diet supplemented with 0.3 mg Se/kg as seleno-yeast; OH-SeMet, basal diet supplemented with 0.3 mg Se/kg as hydroxy-selenomethionine.

The effects of the different forms of Se on selenoproteins’ expression in the jejunum of piglets are shown in Figure 5A. Compared to SeNa, both SeY and OH-SeMet increased (*p* < 0.05) the expression of *GPX6*, while only OH-SeMet increased (*p* < 0.05) the expression of *TXNRD3*. Additionally, only SeY increased (*p* < 0.05) the expression of *GPX3* and *SELENON*, with OH-SeMet showing intermediate values of these genes. In terms of tight junction-related gene expression (Figure 5B), compared to SeNa, only SeY increased (*p* < 0.05) the *ZO-1* expression, with OH-SeMet showing intermediate values. As for host defense peptides (HDPs)-related gene expression (Figure 5C), compared to SeNa, both SeY and OH-SeMet increased (*p* < 0.05) the expression of *pBD1*, *pBD2*, *pBD3*, *NPG3*, and *NPG4*, while only SeY increased (*p* < 0.05) the expression of *PEP2C*. For cytokine gene expression (Figure 5D), compared to SeNa, only SeY increased (*p* < 0.05) the expression of* IL-1β*, *IL-6*, and *IL-10*, with OH-SeMet showing intermediate levels of these genes. Regarding apoptosis gene expression (Figure 5E), compared to SeNa, only SeY decreased (*p* < 0.05) the expression of* Caspase3*, and only OH-SeMet did so for *p53* and *Bcl2*, with OH-SeMet and SeY exhibiting intermediate levels for *Caspase3* and *Bcl2*, respectively.

### 3.7. Production of Selenoproteins and Tight Junction, Cytokine, and Apoptosis Proteins in the Jejunum of Piglets

**Figure 6:**
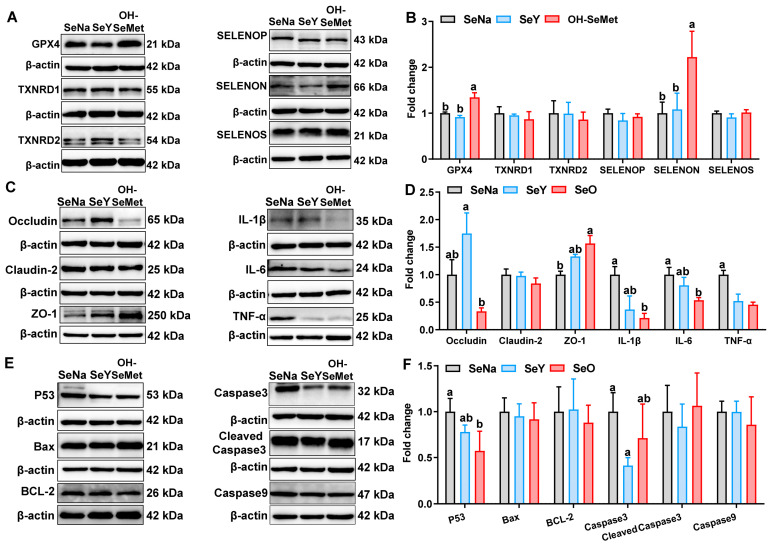
Effects of the three forms of Se on selenoprotein (GPX4, TXNRD1, TXNRD2, SELENOP, SELENON and SELENOS) (**A,B**), tight junction (Occludin, Claudin-2 and ZO-1) and cytokine (IL-1β, IL-6 and TNF-α) (**C,D**), and apoptosis (P53, Bax, BCL-2, Caspase3, Cleaved Cas3 and Caspase9) (**E,F**) protein production in the jejunum of piglets. The values are the means ± SE, *n* = 3–6. Labeled means within the same plot without a common letter differ, *p* < 0.05. SeNa, basal diet supplemented with 0.3 mg Se/kg as sodium selenite; SeY, basal diet supplemented with 0.3 mg Se/kg as seleno-yeast; OH-SeMet, basal diet supplemented with 0.3 mg Se/kg as hydroxy-selenomethionine.

The effects of the different forms of Se on the selenoprotein production in the jejunum of piglets are shown in Figure 6A,B. Compared to SeNa, only OH-SeMet increased (*p *< 0.05) the protein abundance of GPX4 and SELENON. In terms of tight junction and cytokine proteins (Figure 6C,D), compared to SeNa, both SeY and OH-SeMet decreased (*p *< 0.05) the protein abundance of TNF-α. Only OH-SeMet increased (*p* < 0.05) the protein abundance of ZO-1 and decreased (*p *< 0.05) the protein abundance of IL-6, with SeY showing intermediate levels of IL-1β and IL-6. Additionally, compared to SeY, OH-SeMet reduced the protein abundance of Occludin, whereas SeNa showed intermediate levels. As for apoptosis proteins (Figure 6E,F), compared to SeNa, only OH-SeMet reduced (*p *< 0.05) the protein abundance of P53, with SeY showing intermediate levels. Additionally, only SeY reduced (*p *< 0.05) the protein abundance of Caspase3.

## 4. Discussion

Gestation and lactation are states of constant oxidative stress for the sow [7,45], and exposure to a hot environment further exacerbates this stress [4]. Oxidative stress is characterized by increased oxidative damage to lipid, proteins, and DNA [4], leading to suppressed immune function and metabolic disorders [7,8], and consequently poor performance of sows and their offspring. The antioxidant defense is of crucial importance during this physiological and environmental stress of the sow to help her cope with the excessive production of ROS. Selenium (Se), as a key player in the antioxidant system, is essential for maintaining its proper function. The supply of a proper Se source is necessary for supporting the antioxidant system and reducing oxidative damage during stress [46]. Se in the body is found in the form of selenocysteine (SeCys) as part of selenoproteins acting in different levels of the antioxidant system. Dietary Se, once absorbed, is metabolized and converted into a *de novo* SeCys that is immediately inserted into the selenoproteins. Once the requirements for those selenoproteins are met, all the extra Se is excreted to avoid toxicity. However, during these stress situations, there is an increased requirement for selenoproteins to maintain proper antioxidant protection [47,48]. Therefore, the selection of an appropriate selenium source is critical to ensure sufficient selenium availability for selenoprotein synthesis under such conditions. Studies have shown that selenium deposition efficiency of organic selenium sources, such as OH-SeMet and SeY, are superior to inorganic selenium sources like SeNa [10,16,45]. Specifically, OH-SeMet has been demonstrated to have a higher absorption rate and better incorporation into selenoproteins compared to SeY [17,18]. This allows OH-SeMet to more effectively support the antioxidant defense system under stress conditions, as observed in dairy cows [28,29], chickens [17,30,31,32], and swine [33,34].

Given litter birth weight and litter weight gain during lactation also provide an indirect assessment of overall piglet growth performance [49]. The results of litter weight at day 21 and litter body weight gain during days 1–21 in this study imply that an Se supplementation with OH-SeMet could more effectively maintain the offspring growth performance from sows raised under heat stress conditions compared to SeNa or SeY. Of note, the gestation and lactation feed restriction protocol applied in this study followed standard commercial practices and was designed to optimize reproductive performance [37]. This controlled approach minimized variation in maternal body condition across treatments [38], thereby ensuring that the observed effects on offspring growth could be primarily attributed to differences in selenium sources rather than maternal factors. As an antioxidant, Se has been reported to help effectively mitigate the negative effects induced by heat stress in pigs [12] and other animal species [50,51]. In the current study, we compared an inorganic Se source with two organic Se sources and the results imply a better ability of OH-SeMet than SeNa and SeY to maintain the offspring growth performance from sows, as indicated by higher litter body weight gain during 1–21d and a tendency toward an increased number of live-born piglets and those alive at day 21. These results are similar to previous observations, whereby OH-SeMet maintained a better growth performance in broilers [18] than SeNa or SeY, under heat stress conditions, as well as in layers [45] and dairy cows [52]. These findings confirm that the two organic Se sources, OH-SeMet and SeY, are not equivalent in bioavailability, with their SeMet proportion primarily driving efficacy [53].

Adequate Se supplementation can enhance selenoprotein synthesis, thereby more effectively supporting endogenous redox systems [35]. Studies have reported that animals fed OH-SeMet exhibited higher bioavailability than those fed SeY or SeNa, thereby more effectively supporting endogenous redox systems [17,18,33,54]. In the current study, the enhanced endogenous redox systems in sows are supported by enhanced levels of TXNRD and GSH and reduced levels of GSSG in the serum. These findings emphasize the role of organic Se, particularly OH-SeMet, in accumulating in tissues and acting as a storage depot for Se, allowing rapid response to the body’s increased needs for Se and selenoproteins to support endogenous redox systems in challenging conditions such as heat stress [17,18,33].

The effect of the different Se sources on the oxidative stress response also revealed a different ability to regulate immunity, as the immune-inflammatory response is associated with increased oxidative stress [55]. In the current study, the two tested organic Se sources, specifically in the form of OH-SeMet, more effectively enhanced the immune responses compared to SeNa, as evidenced by inflammatory-related markers such as CRP, HP, MAP, LPS, IL-1β, IL-6, and TNF-α, some or all of which were reduced in the serum of sows at the gestation and lactation stages. These results are similar to previous observations where OH-SeMet positively modulated the inflammatory response in pigs under dietary oxidative stress and in broilers under heat stress [18,56]. Cortisol is released in response to different stressors and has been found to limit inflammation, as well as being immunosuppressive, thus inhibiting the production and actions of antibodies [57,58]. The present study demonstrates only OH-SeMet reduced the serum cortisol levels at day 14 of lactation in sows and at day 21 in piglets compared to SeNa. Correspondingly, OH-SeMet was more effective in the production and actions of antibodies, supported by the higher levels of IgG, PED, and TGEV antibodies in the milk of sows. Given the enhanced role of selenium in promoting humoral IgG production, this also implies the higher bioavailability of OH-SeMet [59]. In broilers, Sun et al., [18] also reported reduced levels of IL-6 and higher levels of IgG under heat stress, which are similar to the current study. Overall, these results indicated that OH-SeMet was likely more effective in protecting the health of sows and their offspring through the regulation of immune responses.

Glycolipid metabolism homeostasis is crucial for sows and their offspring, whereas abnormalities in redox status and inflammatory factors could induce disorder of the glycolipid metabolism [60]. As previous studies indicated that OH-SeMet supplementation could alleviate dietary oxidative stress-induced lipid metabolism disorders of pigs [61] and SeMet supplementation could alleviate heat stress-induced glycolipid metabolism disorders in broilers [62], in the current study, we demonstrate that among the two tested organic Se sources, OH-SeMet was more effective than SeNa in maintaining the homeostasis of glycolipid metabolism in sows. This was evidenced by more reduced glucose, TG, and NEFA levels, along with elevated insulin levels in the serum of sows. High blood glucose levels may cause insulin resistance, leading to metabolic disorders such as gestational diabetes [63]; higher serum TG concentrations associated with poor health status of gestating sows [64]; and elevated NEFA, which can potentially impair fertility by altering follicular physiology and reducing oocyte developmental competence [65].

The superior efficacy of OH-SeMet in maintaining the glycolipid metabolism may be attributed to its role in enhancing the antioxidant defense system and modulating immune responses. Selenium, as an essential component of selenoproteins, mitigates oxidative stress and inflammation, both of which are critical drivers of glycolipid metabolism disorders. By improving redox balance and reducing inflammatory damage, OH-SeMet more effectively supports the metabolic and reproductive health of sows, contributing to better offspring growth performance. Here, we also explain why OH-SeMet seemed to more effectively maintain the offspring growth performance of sows.

The gut plays a crucial role in nutrient digestion and absorption, directly contributing to piglet growth [66], as its abnormal development is closely associated with the health status of sows and their milk quality [67,68]. As previously discussed above, OH-SeMet more effectively maintains the health of sows and their offspring. Although the different Se sources did not affect the milk content of fat, protein, lactose, and nonfat solids, the two organic Se sources, particularly OH-SeMet, exhibited higher levels of IgG, PED, and TGEV antibodies in milk compared to SeNa. Correspondingly, OH-SeMet more effectively increased these indicators in the serum or tissue of piglets. In pigs, Se deficiency can seriously affect the health of the small intestine [69], whereas adequate Se enhances intestinal health [70]. In addition, higher levels of PED or TGEV antibodies more effectively protects the intestine against these virus infections, as villous enterocytes in intestine are the major target cells of these viruses [71]. A decrease in crypt depth generally indicates improved gut health and reduced cell turnover in the intestinal lining, while an increased villus-to-crypt ratio is directly associated with enhanced epithelial turnover and nutrient absorption [72]. In the current study, the sows fed the two organic forms of Se, and in particular OH-SeMet, showed a better intestinal morphology and integrity of their offspring, as indicated by a reduced crypt depth and increased villus height/crypt depth ratio in the duodenum, jejunum, and ileum, than those fed SeNa. These results are similar to those of a previous study on broilers [18], where OH-SeMet also demonstrated better gut protection than SeNa under heat stress. Taken together, these outcomes indicate OH-SeMet was more effective than SeNa in protecting the gut integrity of sows’ offspring by maternal influences.

The main function of Se in the body is supporting endogenous redox systems by selenoproteins. In the current study, OH-SeMet has better enhanced endogenous redox systems compared to SeY or SeNa, as shown by improved T-AOC, TXNRD, and GSH alongside decreased MDA and GSSG in the serum of piglets, and heightened T-AOC in the jejunum of piglets. Correspondingly, OH-SeMet exhibited higher expression of *GPX6*, *TXNRD3*, GPX4, and SELENON in jejunum compared to SeY or SeNa. These results suggest that the superior gut protection offered by OH-SeMet is primarily due to its more effective maintenance of endogenous oxidative homeostasis, thereby supporting a better immune response. In the intestine, the disturbance of redox balance may mediate the tight junction disruption and barrier dysfunction [73] and cause abnormal apoptosis of the intestine cells [74]. Here, we found better gut protection of OH-SeMet provided by upregulated protein production of tight junction protein ZO-1 and downregulating pro-apoptotic factor P53 compared to SeNa or SeY. In addition, oxidative stress could induce mucosal inflammation [75] while HDPs can display anti-inflammatory properties [76]. The present study demonstrated that OH-SeMet more effectively enhanced immune responses compared to SeNa, as evidenced by inflammatory related markers such as CRP, HP, MAP, LPS, IL-1β, and IL-6, some or all of which were reduced in the serum of piglets. Furthermore, OH-SeMet downregulated the protein production of inflammation factor IL-6 and IL-1β and upregulated the gene expression of HDPs (pBD1, pBD2, pBD3, NP*G3*, *NPG4*). In summary, OH-SeMet was more effective than SeNa in protecting the gut integrity of piglets by supporting the endogenous redox systems and immune response.

## 5. Conclusions

The results of this study confirm the hierarchy of Se bio-efficacy among different Se sources (SeNa < SeY < OH-SeMet) and suggest Se status may be linked to the increased need for Se for selenoprotein synthesis in challenging conditions such as heat stress. These selenoproteins play a crucial role in responding to oxidative stress, which in turn significantly influences the glycolipid metabolism, and inflammatory and immune responses in animals. OH-SeMet more effectively mitigates the adverse effects induced by heat stress in pregnant sows and supports the better growth performance of their offspring during lactation.

## Figures and Tables

**Table 1:** Effect of the three Se forms on the growth performances of sows and piglets.^1^

Item	SeNa	SeY	OH-SeMet	*p*-Value
**Sows**				
Body weight, kg				
Gestation day 0	224.1 ± 8.9	231.5 ± 6.2	231.9 ± 4.6	0.658
Gestation day 85	257.7 ± 7.0	261.5 ± 4.5	263.1 ± 4.1	0.776
Gestation day 107	278.5+6.8	282.1 ± 4.6	287.0 ± 4.9	0.574
Lactation day 21	252.3 ± 6.6	255.2 ± 4.3	256.7 ± 4.8	0.846
Backfat thickness, mm				
Gestation day 0	18.7 ± 0.6	19.3 ± 0.7	19.4 ± 0.7	0.660
Gestation day 85	19.9 ± 0.9	20.5 ± 0.9	22.6 ± 1.3	0.177
Gestation day 107	20.5 ± 0.9	21.1 ± 1.2	22.8 ± 1.2	0.314
Lactation day 0	20.5 ± 1.0	21.1 ± 1.2	22.6 ± 1.3	0.444
Lactation day 21	18.3 ± 0.9	19.1 ± 0.9	19.7 ± 1.0	0.603
Changes of lactation days 0–21	−2.2 ± 0.4	−2.0 ± 0.7	−2.5 ± 0.5	0.784
Weaning-to-estrus interval, day	3.6 ± 0.3	3.3 ± 0.3	3.3 ± 0.4	0.735
**Piglets**				
Litter size at birth				
Born alive, *n*	12.3 ± 0.5 ^AB^	11.1 ± 0.6 ^B^	12.7 ± 0.5 ^A^	0.095
Stillborn, *n*	0.5 ± 0.2	0.5 ± 0.1	0.7 ± 0.2	0.293
Mummies, *n*	0.1 ± 0.1	0.1 ± 0.1	0.1 ± 0.1	0.789
Alive at day 21, *n*	10.9 ± 0.4 ^ab^	10.2 ± 0.4 ^b^	11.5 ± 0.3 ^a^	0.056
Mortality day 0–21, %	10.48 ± 1.3	9.58 ± 1.3	8.62 ± 1.5	0.635
Diarrhea rate day 0–21, %	3.99 ± 0.7 ^ab^	5.18 ± 0.6 ^a^	3.34 ± 0.5 ^b^	0.050
Piglet’s body weight, kg				
Litter birth weight	18.1 ± 0.9	16.2 ± 0.8	17.4 ± 0.5	0.264
Litter weight at day 21	64.15 ± 3.2 ^b^	62.21 ± 3.4 ^b^	77.65 ± 3.4 ^a^	0.004
Litter body weight gain during days 1–21	46.08 ± 2.7 ^b^	45.53 ± 3.3 ^b^	59.54 ± 3.0 ^a^	0.003
Birth weight/piglet	1.54 ± 0.1	1.48 ± 0.1	1.44 ± 0.1	0.389
Body weight/piglet at day 21	6.18 ± 0.2	6.14 ± 0.3	6.72 ± 0.2	0.138
Body weight gain/piglet during days 1–21	4.63 ± 0.2	4.65 ± 0.3	5.29 ± 0.2	0.124

^1^ The values are the means ± SE, *n* = 19–20. Labeled means within the same plot without a common lowercase letter indicate significance at *p* < 0.05; a common uppercase letter indicates a significance level of 0.05 ≤ *p* < 0.10. SeNa, basal diet supplemented with 0.3 mg Se/kg as sodium selenite; SeY, basal diet supplemented with 0.3 mg Se/kg as seleno-yeast; OH-SeMet, basal diet supplemented with 0.3 mg Se/kg as hydroxy-selenomethionine.

## Data Availability

Data are contained within the article.

## Supplementary Materials

The following supporting information can be downloaded at https://www.mdpi.com/article/10.3390/antiox14050525/s1, Table S1: Ingredients and chemical composition of basal diets (as-fed basis, %); Table S2: List of primers used for Real Time-qPCR analysis; Table S3: Name, type, dilution, and source of primary antibodies; Figure S1: Daily mean temperature–humidity index of the environment; Figure S2: The effects of the three forms of Se on milk composition.
