# Supplementary material for: Hydroxy-Selenomethionine Supplementation During Gestation and Lactation Improve Reproduction of Sows by Enhancing the Antioxidant Capacity and Immunity Under Heat Stress Conditions
Source: Antioxidants (Basel). 2025 Apr 27;14(5):525. doi: 10.3390/antiox14050525 (PMC12108442; doi:10.3390/antiox14050525)
Supplement: Supplementary file 1 [file antioxidants-14-00525-s001.zip › antioxidants-3505667-supplementary.pdf]

Supplemental Table S1. Ingredients and chemical composition of basal diets (as-fed basis, %)

Ingredients, %	Gestation	Lactation
Corn	30.8	38.2
Soybean meal	10.25	20.8
Broken rice	16	16
Rice bran meal	6.0	-
Fish meal	-	2.0
Wheat bran	16.0	-
Wheat germ	-	10
Soybean hull	10.0	-
Fiber ingredient	1.0	1.0
Soybean oil	1.0	3
CaHPO4	1.3	1.3
CaCO3	1.0	0.9
Fermented feed ingredients	4.5	4.5
NaHCO3	0.25	0.25
L-Lys, 70%	0.25	-
L-Met, 98%	-	0.4
Salt	0.5	0.5
Choline chloride	0.15	0.15
Premix[1]	1.0	1.0
Total	100	100
Nutrient content, %		
Digestible energy, MJ/kg	12.21	13.90
Net energy, MJ/kg	9.37	10.58
Crude protein, %	13.66	17.97
Crude fiber, %	7.87	3.95
Calcium, %	0.81	0.81
Total phosphorus	0.62	0.66

[1] The analysed selenium in the diets of SeNa, SeY and SeO during gestation are 0.352, 0.337, and 0.364 mg/kg, respectively, and during lactation are 0.374, 0.397, and 0.386 mg/kg, respectively.

[2] Premix provided the following per kilogram of basal diet: vitamin A, 8,000 IU; vitamin D3, 2,000 IU; vitamin E, 12.5 IU; vitamin K 2.5 mg; Biotin, 0.2 mg; folacin, 0.25 mg; niacin, 17.5 mg; pantothenic acid, 12.5 mg; riboflavin, 8.0 mg; thiamin, 1.0 mg; vitamin B6, 3.00 mg; vitamin B12, 15 µg; copper, 16 mg; iodine, 0.3 mg; iron, 165 mg; manganese, 30 mg; zinc, and 165 mg. The sources of the trace elements were CuSO4·5H2O, KI, FeSO4, MnSO4·H2O, and ZnSO4, respectively.

Supplemental Table S2. List of primers used for Real Time-qPCR analysis[1]

	Accession number	Forward primer (5′to 3′ direction)	Reverse primer(5′to3′direction)
ZO-1	XM_032391268.1	GCCAGCTGGAGCTTAGAACA	GGCATCAAGAGGGGCTACTG
Occludin	NM_001163647.2	CAGGTGCACCCTCCAGATTG	ATGTCGTTGCTGGGTGCATA
Claudin 1	NM_001161635.1	GCCACTGTTGGCATGAAGTG	CTGGCATTGACTGGGGTCAT
Claudin 2	XM_021079578.1	ATCCTCTGCTTTTCCTGCCC	CTGTCCACTGGCTCTCACAG
pBD1	NM_214442.2	CCAGCTGGCTGCAGGTATTA	ACTTGGCCTTGCCACTGTAA
pBD2	NM_213738.1	AACCTGCTTACGGGTCTTGG	TTGCCACTGTAACAGGTCCC
pBD3	XM_021074698.1	CACGCCTTCCTATCCAGTCT	CATTTCCTCTTTCGGCAGCA
PG1-5	XM_021070622.1	GTAGGTTCTGCGTCTGTGTCG	CAAATCCTTCACCGTCTACCA
PEP2C	XM_003362076.4	ACCAAGGGTCCCAGAGAACT	GGCTCTGGAGAACAAGCAGT
NPG1	NM_001123149.1	TGACTTCAAGGAGAACGGGC	GGAACCTTGGTGGACCGAAT
NPG3	NM_001123150.1	TGACTTCAAGGAGAACGGGC	ACAATAGCACAGGCCACCTC
NPG4	NM_001123150.2	CGGAGCTGTGTGACTTCAAG	AACCGTGGTCTTCGTAGGAA
IL-1β	NM_214055.1	GCCCAATTCAGGGACCCTAC	GGCGGGTTCAGGTACTATGG
IL-2	XM_021100436.1	CCCTTGCACTCATGGCAAAC	TAGCACTCCCTCCAGAGCTT
IL-6	NM_214399.1	GCAGTCACAGAACGAGTGGA	CTCAGGCTGAACTGCAGGAA
IL-8	NM_213867.1	CTTCCAAACTGGCTGTTGCC	GTTGTTGTTGCTTCTCAGTTCTCT
IL-10	NM_214041.1	AGAGGGGTGTCTACAAAGCC	AGAGGTACAGCAGGGTTTCC
TNF-α	NM_214022.1	GGCCCAAGGACTCAGATCAT	CTGTCCCTCGGCTTTGACAT
Caspase3	NM_214131.1	TCTAAGCCATGGTGAAGAAGGAAAAA	CCCCTCTGAAGAAACT
Caspase9	XM_013998997.2	CCGATTTGGCTTACGTCCTG	CAAAGCCTGGACCATTTGCT
P53	NM_213824.3	GCCGCACAGAGGAAGAAAAT	CAAGGCGTCATTCAGCTCTC
BCL-2	XM_064578768.1	CTTCAGGGATGGGGTGAACT	GCCCATACAGCTCCACAAAG
Bax	XM_013998624.2	TGGACTTCCTTCGAGATCGG	CCTCAGCCCATCTTCTTCCA
β-actin	XM_021086047.1	CTACACCGCTACCAGTTCGC	AGGGTCAGGATGCCTCTCTT

[1]ZO-1, zonula occludens-1; pBD1, 2 and 3, Porcine β-Defensin 1, 2 and 3; PEP2C, epididymis protein 2 splicing variant C; NPG1, 3 and 4, protegrin 1, 3 and 4; IL -1β, -2, -6, -8 and -10, Interleukin -1β, -2, -6, -8 and -10; TNF-α, tumor necrosis factor-α; P53, tumor protein p53; BCL2, B-cell lymphoma-2; Bax, B-cell lymphoma-2 associated X.

Supplemental Table S3. Name, type, dilution, and source of primary antibodies[1]

Antibody	Isotype	Dilution	Source
Primary antibody			
GPX4	Rabbit	1:1000	ABclonal (Wuhan, China)
TXNRD1	Rabbit	1:1000	Abcam (Cambridge, MA)
TXNRD2	Rabbit	1:1000	Proteintech (Wuhan, MA)
SELENOP	Rabbit	1:1000	Abcam (Cambridge, MA)
SELENON	Rabbit	1:1000	ABclonal (Wuhan, China)
SELENOS	Rabbit	1:1000	ABclonal (Wuhan, China)
Occludin	Rabbit	1:1000	ABclonal (Wuhan, China)
Claudin-2	Rabbit	1:1000	ABclonal (Wuhan, China)
ZO-1	Rabbit	1:1000	ABclonal (Wuhan, China)
IL-1β	Rabbit	1:1000	ABclonal (Wuhan, China)
IL-6	Rabbit	1:1000	ABclonal (Wuhan, China)
TNF-α	Rabbit	1:1000	ABclonal (Wuhan, China)
P53	Rabbit	1:10000	ABclonal (Wuhan, China)
Caspase3	Rabbit	1:1000	ABclonal (Wuhan, China)
Cleaved-caspase3	Rabbit	1:1000	ABclonal (Wuhan, China)
BCL-2	Rabbit	1:1000	ABclonal (Wuhan, China)
β-actin	Rabbit	1:10000	ABclonal (Wuhan, China)
Secondary antibody			
HRP Antibody	Goat anti rabbit	1:10000	ABclonal (Wuhan, China)

[1]GPX4, glutathione peroxidases 4; TXNRD1, 2, thioredoxin reductase 1, 2; SELENOP, N, S, selenoprotein P, N, S; ZO-1, zonula occludens-1; IL-1β, -6, Interleukin -1β, -6; TNF-α, tumor necrosis factor-α; BCL-2, B-cell lymphoma-2.

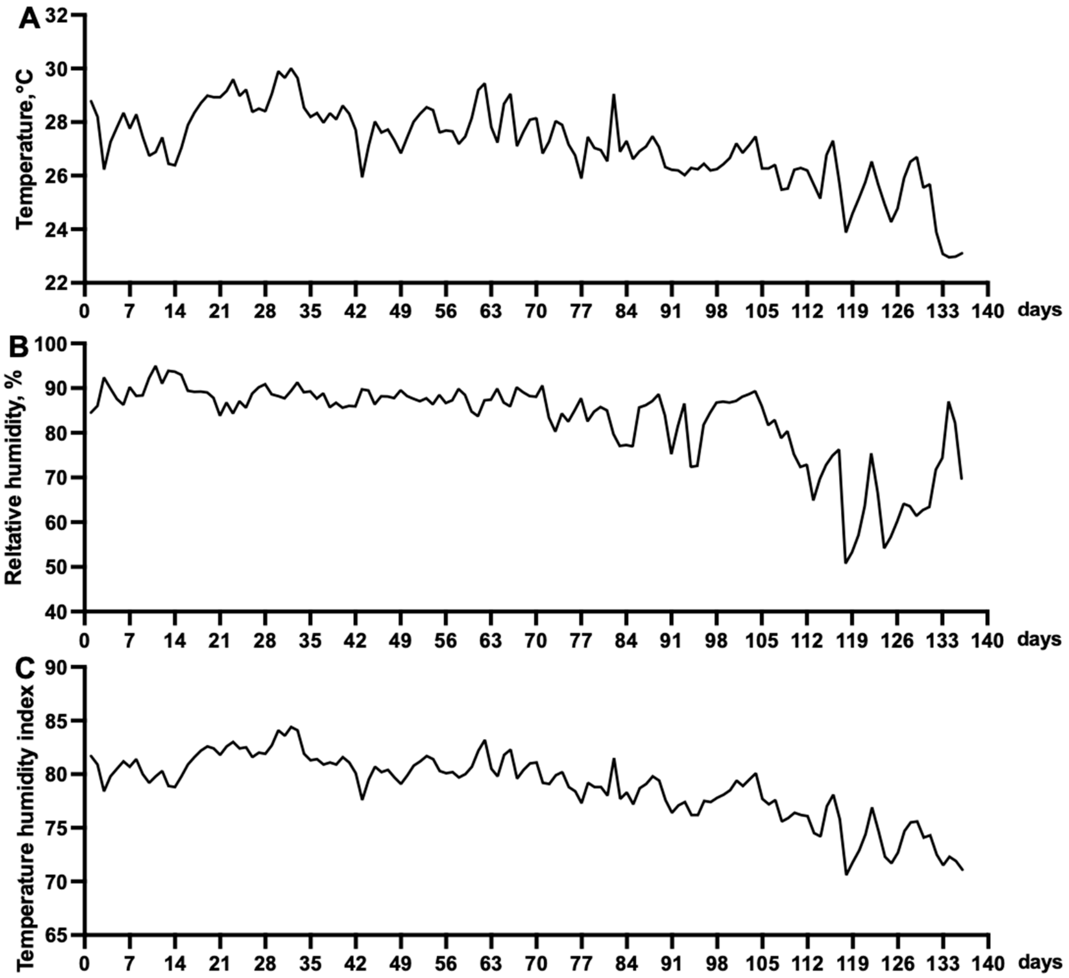

Supplemental Figure S1. Daily mean temperature (A), relative humidity (B) and temperature-humidity index (C) of the environment. The data was collected from 07:00 to 19:00 over the 135 days of the experiment. Temperature-humidity index calculated as THI = (1.8 × daily average temperature+ 32) − [(0.55 − 0.0055 × daily average relative humidity) × (1.8 × daily average temperature − 26)]. During the 135-day period, the average temperature, humidity, and temperature-humidity index ranged from 22.9–33°C, 50.8%–94.9%, and 70.6–84.4, respectively.

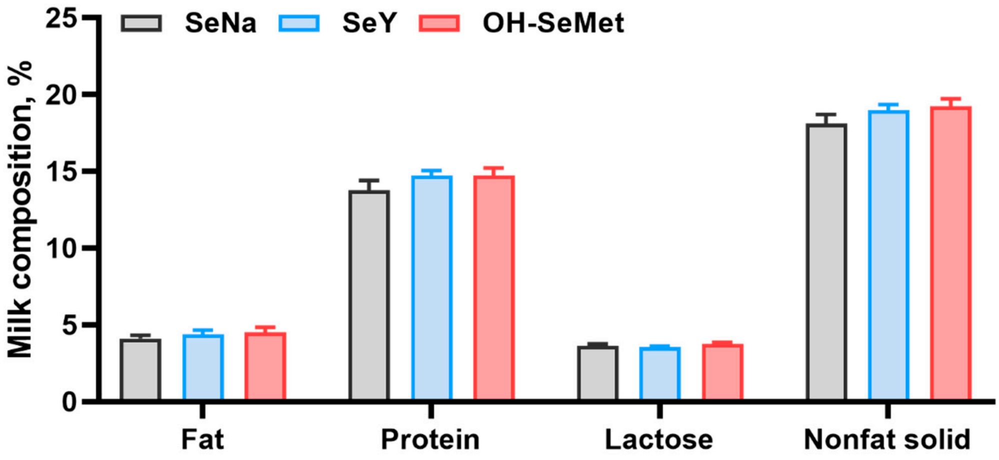

Supplemental Figure S2. The effects of the three forms of Se on milk composition. The values are the means ± SE, n = 19-20. Labeled means within the same plot without a common letter differ, $P < 0.05$. SeNa, basal diet supplemented with 0.3 mg Se/kg as sodium sele-nite; SeY, basal diet supplemented with 0.3 mg Se/kg as seleno-yeast; OH-SeMet, basal diet supplemented with 0.3 mg Se/kg as hydroxy-selenomethionine.
